# Information Needs of Patients With Head and Neck Cancer and Their Supports in Relation to Treatment Management Choices: Scoping Review

**DOI:** 10.2196/64108

**Published:** 2025-08-21

**Authors:** Eleah Stringer, Lily Hallett Rio, Lorraine Leitz, Eitan Prisman, Elizabeth Borycki, Andre Kushniruk, Jonathan Livergant, Sally Smith

**Affiliations:** 1 School of Health Information Science, University of Victoria, Victoria, BC, Canada; 2 BC Cancer – Victoria, 2410 Lee Ave, Victoria, BC, V8R 6V5, Canada, 1 250 519 5523, 1 250 519 5721; 3 Department of Surgery, University of British Columbia, Vancouver, BC, Canada

**Keywords:** head and neck cancer, information needs, collaborative decision-making, patient-centered care, decision aid, decision support, user needs, cancer, treatment management, information, scoping review, medical information, patient information, treatment, patient, caregiver, support, decision-making

## Abstract

**Background:**

Advances in research and modes of information delivery provide new opportunities to access medical information. Despite this, patient information needs on head and neck cancer (HNC) treatment are not sufficiently met.

**Objective:**

The aim is to investigate (1) information content required for patients with HNC and their caregivers to support confident decisions about their treatment, (2) information needs by role (eg, patient and caregiver), and (3) the preferred format or mode of information delivery. Results will be used to inform the development and testing of a decision aid for this patient population.

**Methods:**

A scoping review was conducted using the Arksey and O’Malley and Levac et al frameworks. The search was carried out in CINAHL, MEDLINE, Embase, and Cochrane Central Register of Controlled Trials and limited to the English language between 2012 and the search date of September 20, 2022. Studies were dual-screened against inclusion and exclusion criteria, central to which was a focus on information needs within the context of decision-making. Data were extracted from the articles using prespecified criteria into a data extraction sheet that was pilot-tested and refined prior to its application. Reporting followed the research questions and was guided by PRISMA-ScR (Preferred Reporting Items for Systematic Reviews and Meta-Analyses Extension for Scoping Reviews).

**Results:**

A total of 10,495 publications were identified, with 30 articles suitable for data extraction. High information needs included details of the diagnosis (3/30, 10%), purpose (6/30, 20%), and risks (10/30, 33%) of medical procedures; strategies for eating and speaking during and after treatment (6/30, 20%); lifestyle guidelines for survivorship (4/30, 13%); and facts about the human papillomavirus (2/30, 7%). Moderate information needs included the physical (10/30, 33%) and psychological (17/30, 57%) domains of posttreatment, treatment options (6/30, 20%), strategies to improve communication with health care providers (8/30, 27%), and nutrition (8/30, 27%). Information needs of patients with HNC and their caregivers evolved through the phases of treatment, highlighting the need for relevant information to support collaborative decision-making with their health care providers. Caregiver needs were underrepresented (5/30, 17%), but more information on stress reduction strategies for the patient, how to play a role in treatment decisions, and where to obtain the best medical care for the patient was identified. The preferred mode of delivery for information varied and reflected the age, gender, and country of the sample populations.

**Conclusions:**

Information needs of patients with HNC and their caregivers are not being met to a satisfactory level, evidenced by the breadth of outstanding needs. Health care providers must consider evolving patient and caregiver information needs, addressing concerns on an individual basis to support shared decision-making. Tools are needed to support information delivery that is acceptable to patients and caregivers.

## Introduction

It is estimated that 7500 Canadians were diagnosed with head and neck cancer (HNC) in 2022, with 28% of those individuals losing their lives to the disease [1]. The incidence of HNC is increasing globally and is projected to continue to rise, with a predicted 30% increase annually by 2030 [2,3]. Approximately 90% of HNCs originate from squamous cells of the epithelial lining in the oral cavity, pharynx, and larynx, but can also arise from salivary glands, sinuses, and muscles or nerves in the head and neck region [1,2]. Major risk factors for HNC include the use of tobacco, alcohol, or betel quid and areca nut (ie, chewing paan), as well as the human papillomavirus (HPV), especially HPV type 16 in oropharyngeal cancers. There is wide global variation in the rates of HPV-associated HNC, but the majority of cases are expected to be HPV positive within the next 20 years, which is significant because the risk of mortality drops nearly 60% in these cases [2,3].

Patients with HNC may have an option of selecting between various treatment modalities, depending on the cell type, tumor, staging, and presentation [4]. It can be distressing and difficult for patients to delineate the options and make a decision that best aligns with their goals and values. Deciding on cancer care requires the provision of correct and sufficient information in a manner that is understandable by the patient.

While significant advancements and health care policy rooted in client- and family-centered care through which “making space for mutual understanding” is a core construct [5], the patient voice continues to highlight that information needs are still not fully met in our standard model of verbal information delivery [6]. Meeting the patient’s information needs has been demonstrated to increase patient confidence in medical decision-making while also improving quality of life (QOL). A 2010 systematic review on the relationship between information provision and health-related QOL discovered all 5 prospective observational studies included in their results to have “found a positive relationship between appropriate information provision (satisfaction with the received information, fulfilled informational needs, high quality and clear information) and mental and global health-related quality of life and a negative relationship between appropriate information provision and depression and anxiety” [7].

In a previous project, our research team carried out patient-oriented, semistructured interviews with survivors of HNC, who completed treatment at BC Cancer – Victoria. In this research, we studied cancer care experiences, learned about the theoretical use of a patient decision aid, and developed insights into the ideal design features of a decision aid [8]. Patients indicated that they were supportive of using mobile app–based visualizations. Such visualizations were viewed as increasing the transparency and comprehensibility of information on treatment options to help patients engage in collaborative decision-making with health care professionals. We are now working with a group of survivors of HNC to co-develop a decision aid in the form of an app to be used on an iPad or other electronic device. To help inform this process, we conducted a scoping review to explore and synthesize the relevant literature. Specifically, the research questions were as follows: (1) What do patients with HNC and their supports need or want to know to help them make a confident and informed decision on cancer treatment options? (2) What are their information needs by role (eg, patient and caregiver)? (3) What are the preferred modes of information delivery to inform the development and testing of a decision aid for patients with HNC?

## Methods

A scoping review was conducted using the Arksey and O’Malley [9] and Levac et al frameworks [10] to answer the research questions. Articles must focus on information needs within the context of decision-making; articles on information needs but excluding decision-making were not included. The full inclusion and exclusion criteria are summarized in Textbox 1. The search method was developed around the search terms in Textbox 2 and in consultation with a medical librarian with expertise in cancer (see full search strategy in Multimedia Appendix 1). The search was limited to articles published in English since 2012 and in adults. The searches were run on September 20, 2022, in CINAHL, Ovid MEDLINE, Ovid Embase, and Ovid Cochrane Central Register of Controlled Trials. Forward searching was conducted on the included articles. Results were exported in Covidence [11], duplicates removed, and article titles and abstracts were screened by 2 researchers, among ES, LHR, LL, and EB, independently. Conflicts were resolved by discussion and a unanimous decision involving a third researcher, AK. Each article was then read in depth. Articles were excluded if, upon further review, the article did not meet the study criteria (see Textbox 1).

A data extraction sheet was created a priori. Data were extracted from the articles using prespecified extraction criteria. The data extraction sheet and process were pilot-tested and refined prior to their application to all of the articles. Inductive analysis was applied to identify themes and patterns within the extracted data. Researchers ES and LHR coded the extracted data and grouped codes to form emerging themes. Themes were presented, discussed, and decided upon unanimously by the research team. Reporting followed the research questions and was guided by PRISMA-ScR (Preferred Reporting Items for Systematic Reviews and Meta-Analyses Extension for Scoping Reviews). The scoping review protocol was not published.

Textbox 1. **Inclusion and exclusion search criteria.**

**Inclusion criteria**

- Publication type: academic, peer-reviewed original research (the following methodologies will be included in the search but filtered out for background information: original research, systematic reviews, scoping reviews, narrative reviews)
- Written in English or translated into English
- Publication from 2012 to present
- Population of focus is patients and survivors of head and neck cancer (including salivary glands and nasopharyngeal carcinoma), their family, and other supports (eg, formal and informal caregivers)
- Focuses on educational needs related to choices on head and neck cancer treatment and management (surgery, systemic therapy, radiation, and combinations thereof)

**Exclusion criteria**

- Publications that are not academic, peer-reviewed resources (eg, gray literature)
- Information needs are not related to treatment choices (eg, supportive care information needs such as side effect management)
- Population: multidisciplinary care team perspectives and cancers of the thyroid
- Interventions related to info delivery, needs, care pathways, apps, etc
- Creating or testing an intervention

Textbox 2. **Core search terms.**

**Patients with head and neck cancer or their caregivers**

- Head and neck
- Oropharyngeal
- Cancer
- Carcinoma
- Neoplasm
- Patients
- Survivors
- Spouses
- Family
- Support persons
- Friends
- Caregivers

**Information seeking**

- Information needs
- Information seeking
- Information dissemination
- Information sources
- Access to information
- Information services
- Health communication
- Teaching materials
- Health education
- Decision-making

**Treatment and management**

- Cancer treatment
- Cancer management
- Radiation therapy
- Chemotherapy
- Therapy
- Systemic therapy
- Immunotherapy
- Surgery
- Transoral robotic surgery

## Results

### Study Selection

**Figure 1.**
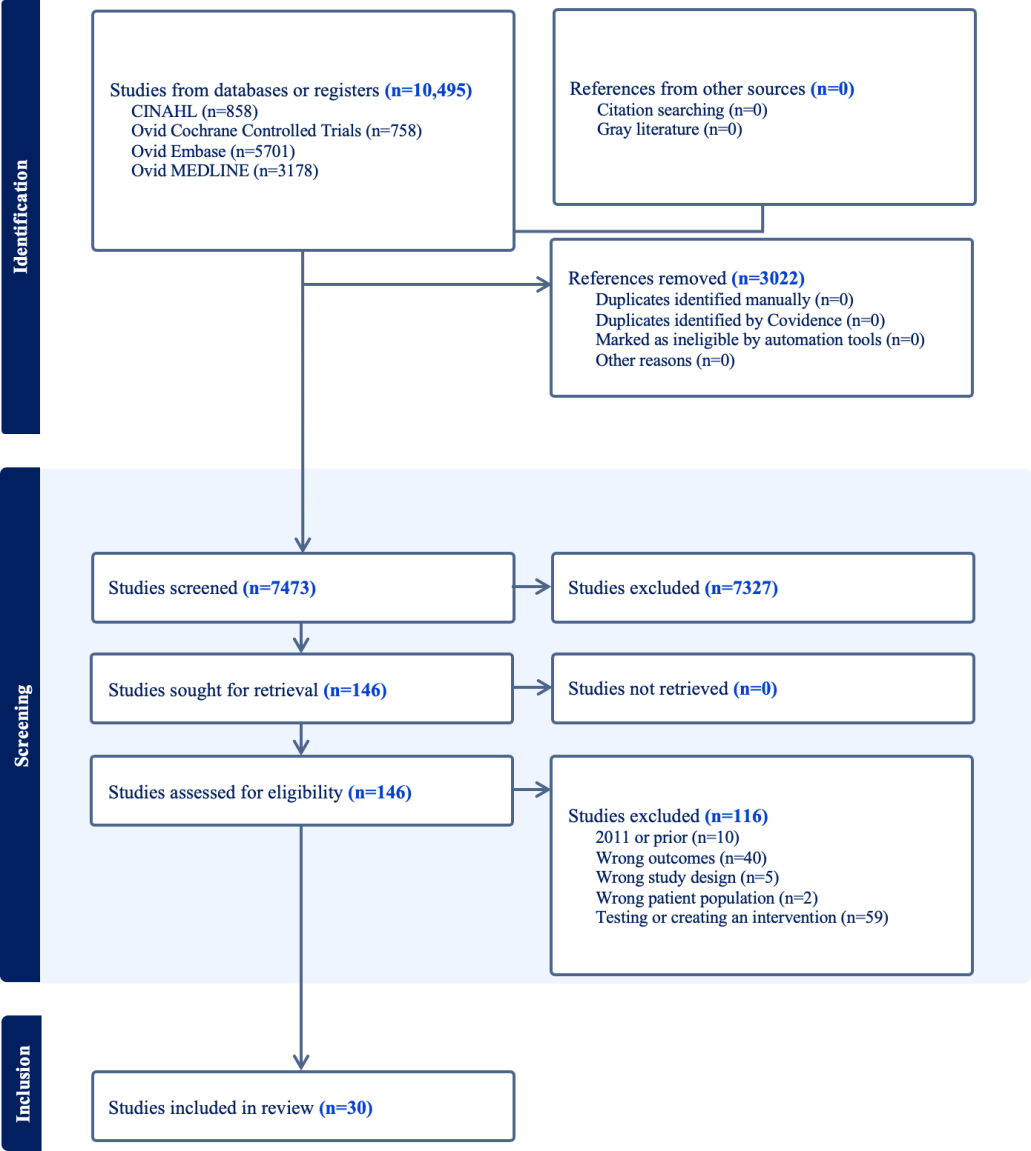
PRISMA (Preferred Reporting Items for Systematic Reviews and Meta-Analyses) diagram of article selection.

A total of 10,495 articles were identified, with 3022 duplicates removed, leaving 7473 articles that underwent title and abstract screening. Of these, 146 articles underwent a full text review, which resulted in 30 final articles from which data were extracted. See Figure 1 for a PRISMA diagram.

### Study Details and Characteristics

The characteristics of the articles are described in Table 1. Forty percent (12/30) of the articles were from the United States and Canada, with others from Europe, Australia, and South Asia, with 83% (25/30) of the articles published between 2015 and 2021. The vast majority (21/30, 70%) of the articles focused on information needs during the posttreatment, survivorship phase. The perspective of male patients dominated this literature. Of the 30 articles, 13 (43%) used a cross-sectional study design, followed by 9 (30%) qualitative interviews. The articles discussed a variety of individual and multimodality treatments. The research settings varied, and diagnostic details (eg, location, staging, and type of HNC) were only specified in a handful of studies; therefore, information needs were pooled across studies rather than stratified by diagnosis or research setting.

**Table 1.** Article characteristics, n=30.

Category and subcategory	Values, n (%)
Country	
United States	8 (27)
Canada	4 (13)
The Netherlands	4 (13)
Australia	4 (13)
Taiwan	3 (10)
England	2 (7)
Scotland	2 (7)
Ireland	1 (3)
France	1 (3)
Multiple	1 (3)
Publishing year	
2012	1 (3)
2013	1 (3)
2014	2 (7)
2015	3 (10)
2016	6 (20)
2017	3 (10)
2018	5 (17)
2019	1 (3)
2020	3 (10)
2021	4 (13)
2022	1 (3)
Trajectory phase	
Pretreatment	1 (3)
Posttreatment	21 (70)
Multiple time points	1 (3)
Varied	5 (17)
At recurrence	1 (3)
Not available	1 (3)
Gender of participants (majority)	
Male	24 (80)
Female	5 (17)
Not specified	1 (3)
Population of intended material	
Patient	25 (83)
Caregiver	3 (10)
Patients and caregivers	2 (7)
Study design	
Prospective	2 (7)
Cross-sectional	13 (43)
Retrospective	1 (3)
Longitudinal	2 (7)
Qualitative research^a^	9 (30)
Mixed methods	1 (3)
Not specified by authors	2 (7)
Primary treatment	
Surgery alone	6 (20)
Surgery + radiation	3 (10)
Surgery + chemo/radiation	3 (10)
Radiation alone	6 (20)
Chemoradiation alone	3 (10)
Multimodality (not otherwise specified)	3 (10)
Not provided by authors	6 (20)

a Defined as one or both of interviews and focus groups.

### Information Needs for Treatment Decisions

#### Priority of Information Needs

Through inductive analysis, information needs were prioritized into high, medium, or low information needs as listed in Table 2. The categorization reflected the number of articles, as well as the proportion of participants in each article that indicated a need. Needs were categorized as high when 55%‐100% of study participants indicated a need, moderate when 21%‐54% of study participants indicated a need, and low when 20% or less of study participants indicated a need.

**Table 2.** Prioritization of information needs, n=30.

Information need	Articles where need indicated, n (%)
High-priority needs^a^	
Disease information (prognosis, stage, symptoms)	12 (40)
Benefits and side effects of treatment	10 (33)
How to live a healthy and improved lifestyle posttreatment	8 (27)
Strategies to improve speaking and eating	6 (20)
Medical tests	6 (20)
Symptoms of recurrence	3 (10)
Reducing stress in patients’ lives	3 (10)
How to play a role in treatment decisions	2 (7)
Obtaining the best medical care for the patient	2 (7)
Human papillomavirus transmission	2 (7)
Moderate-priority needs^b^	
Physical and daily living (QOL^c^) needs posttreatment	14 (47)
Coping with stress and anxiety	11 (37)
Availability of support services	10 (33)
Communicating with health care professionals	8 (27)
Nutrition	8 (27)
Long-term side effects of treatment	6 (20)
Appearance and body image concerns	6 (20)
Coping with fear of recurrence	6 (20)
Strategies for managing social situations	6 (20)
Treatment options	6 (20)
Coping strategies	5 (17)
Changes in eating and speaking	5 (17)
Strategies for pain and fatigue management	4 (13)
Communicating with family members	4 (13)
Head and neck specific needs (side effects and education)	3 (10)
Supportive care information	3 (10)
Obtaining or maintaining insurance postcancer	2 (7)
Looking after caregivers’ health	1 (3)
Coping with fears about patients’ deterioration	1 (3)
Balancing caregiver and patient needs	1 (3)
Low-priority needs^d^	
Financial and work impact	10 (33)
Intimacy and sexuality health	7 (23)
Oral care	3 (10)
Fertility after treatment	2 (7)

a 100%-55% of participants indicated a need.

b 54%-21% of participants indicated a need.

c QOL: quality of life.

d 20%-0% of participants indicated a need.

#### High Needs

##### Disease Information (Prognosis, Stage, and Symptoms)

Conventional areas of pretreatment medical information were commonly covered in pretreatment surgical and oncological consultations. This includes, but is not limited to, the disease process, recommended treatment options, and prognosis [12]. Generally, patients found this information suitable and substantial [12-14], although satisfaction was not consistent, as Jehn and colleagues [15] noted that some patients felt the pretreatment information was inadequate. Specifically, information needs on prognosis were explicitly listed as not met in 3 (10%) out of the 10 studies [12,16,17], with patients indicating the crucial and foundational nature of this information to their decision-making [18,19]. Hoesseni and colleagues [18] noted that while patients were provided with prognostic information, such as life expectancy, they were unfamiliar with the concept, found it confusing, or had a negative interpretation of the information. In a study conducted in Taiwan, Chen and colleagues [16] report on the time constraints otolaryngologists face, hindering their ability to discuss a patient’s condition with the caregiver, which may contribute to unmet prognostic needs. Additionally, patients indicated that they wanted information on both their specific type and stage of disease as well as general information about cancer [12,17,19-22].

##### Side Effects of Treatment and Decision-Making

In 10 (33%) of the 30 articles, information on the side effects of treatment was indicated as a high information need [12,16,17,19,20,23-27]. Participants considered information on the possible side effects of treatment to be very important [17,19]. Three (10%) articles reported on the desire to learn more about the short-term side effects of treatment [20,23,24], with 80% (n=41) of the patients surveyed by Berkowitz and colleagues [20] reporting this as an information need. Robust information on potential advantages and side effects of treatment modalities was important for patient decision-making on treatment choice [25,26]. A small study (n=12) found, when information was not adequately provided, patients were left with a sense of mistrust [27].

##### Medical Tests

In 6 (20%) of the 30 articles, information on medical tests was cited as a need [17,20-22,25,26]. Over 50% (114/205) of patients noted that more information was needed regarding tests and follow-up investigations [17]. Berkowitz and colleagues [20] found that 38 out of 39 (97%) patients indicated that written information describing tests that are mandatory on a recurring, long-term basis would have been helpful. Additionally, information about how to prepare for tests was noted as moderately important, although the specifics of this information were not provided [17]. Jansen and colleagues [25] found that a small proportion of patients wanted more thorough explanations of the tests, as well as quicker communication of the results.

##### How to Live Healthy and Improved Lifestyle Posttreatment

Strategies to live a healthy lifestyle during and following the completion of treatment were frequently cited as an information need [12,17,20,28]. Berkowitz and colleagues [20] noted almost that all participants understood the importance of a healthy diet and physical activity on recovery; however, 35 of 39 (90%) patients endorsed the statement, “I am interested in learning more about ways to stay healthy after my cancer diagnosis and treatment,” in survey responses. This need was also identified in 2 (7%) articles by a smaller subset of patients but was instead broadly framed as information to help oneself get well [25,26].

##### Strategies to Improve Speaking and Eating

In 6 (20%) of the 30 articles, strategies to improve speaking and eating were noted [12,17,19,23,26,28]. Papadakos et al [19] reported that 69.3% (n=450) of their participants felt it was important to have information on eating, drinking, and managing pain when swallowing. This finding is supported by the work of Badr and colleagues [23] and Saroa and colleagues [17], who reported that 81% (n=93) and 58% (n=205) of participants, respectively, desired information on strategies to improve speaking and eating. Three (10%) other studies supported this finding in a moderate capacity [12,26,28]. Although a lower priority, 2 (7%) articles also indicated patients had needs relating to managing loss of taste and difficulty breathing [25,29].

##### Symptoms of Recurrence

Three (10%) of the 30 articles noted that patients needed education on symptoms of recurrence [17,19,26]. Manne and colleagues [26] noted that more than half of the participants (n=92) retroactively reported they did not receive a written summary of the recommended follow-up care, including information about active surveillance for suspicious lesions. This need was more common among patients who had experienced a recurrence or had a greater fear of recurrence.

##### HPV Transmission

In 2 (6.7%) of the 30 articles, HPV in relation to HNC was addressed [19,30]. Papadakos and colleagues [19] found that patients had information needs relating to HPV transmission. Their study found that 303 (67.4%) of their participants needed more information on whether their partners could contract or develop HPV-related cancer. Inglehart and colleagues [30] conducted a health literacy analysis among HPV-positive and HPV-negative patients with cancer. Among HPV positive cases (n=187), significant knowledge gaps were identified despite adequate health literacy. Only 56% (n=105) of these patients felt they had enough knowledge to discuss HPV with their physician or partner.

### Moderate Needs

#### Physical and Daily Living (QOL) Needs Posttreatment

The most frequently cited moderate information needs fell into the categories of physical (10/30, 33%) and psychological health (17/30, 57%) posttreatment. This encompassed topics related to the management of side effects and pain (2/30, 7%) [31,32], performing daily tasks and adjusting to a new QOL (7/30, 23%) [18,22,25-27,31,33], accessing information on how to care for patients (1/30, 3%) [16], and the management of medical routines at home (4/30, 13%) [17,22,25,34]. A small study (n=11) indicated that despite pretreatment consultations regarding medical management with health care professionals, patients felt underprepared for the impact of treatment on their lifestyle [35]. This need was echoed in a large study (n=1359), where 37.1% (n=504) of patients retrospectively indicated a lack of information regarding the potential physical and psychological consequences of surgery [15]. Information regarding the impact on QOL was cited as an important factor in treatment decisions [18]. Jansen and colleagues [25] noted that unmet health information and support needs were more prevalent among patients who lived alone.

#### Psychological Needs

Psychological needs were prevalent in the results, mentioned in 17 (57%) out of the 30 articles. Broadly, there was a gap in the dissemination of available support services to patients. Patients required more information on accessing support groups, professional counseling, and health care and social services [12,17,21,25-28,31,36,37]. Articles specifically indicated information was needed for stress and anxiety management [12,17,22,23,25-29,31,33], appearance and body image concerns [12,17,23,26,28,31], dealing with fears of recurrence [22,24,26,29,31,33], strategies for managing social situations, such as eating in public [17,23,25,26,28,31], and pain and fatigue management [16,17,23,29]. Participants desired more information on coping strategies, such as keeping a positive mindset, seeking social support, and using religious beliefs and practices [17,25,28,31,33].

#### Communicating With Health Care Professionals

Strategies to assist patients in improving dialogue and conversation between themselves, physicians, and nurses were a moderate need in 8 (27%) of the 30 articles. This manifested as a need for both improved patient-provider communication [22,32] and provider-provider communication [31,36]. A study of 59 caregivers found that over half of the study group did not know what questions to ask health care providers [38]. Similarly, Parker and colleagues [27] also found that a portion of their 12 participants did not know what questions to ask health care professionals.

Evidence of communication between health care professionals was important for participants to feel secure in the coordination of their care plan [31]. Patients indicated that this communication was important to ensure a seamless transition between the hospital and the home. Patients felt that their general practitioner should be more involved and well informed of their medical status by other health professionals [36]. Similarly, the review found that more resources to support and enhance communication between patients and family members were needed. Articles identified that patients wanted strategies to better communicate with family members to alleviate and manage psychosocial concerns [23] and that this need was more prevalent among those with early-stage disease compared to late-stage disease [28]. Work by van Overveld and colleagues [36] found that patients also desired resources on how to involve children in the health care process.

#### Long-Term Side Effects of Treatment

Generally, more information was needed on the long-term effects of treatment [12,20,26], specifically the anticipated duration of persistent symptoms [12,39]. Patients described feeling underprepared for the severity and longevity of symptoms, causing patients to experience “a reality of uncertainty” [40]. Patients thought symptoms would resolve faster, but by some, the course of recovery was perceived as slow [33]. Patients were told to expect a long recovery but identified discrepancies between the information provided by health care professionals and how they interpreted the information [33]. For some patients, this disconnect led them to “doubt and question both the verbal and written information they had received” [40].

#### Treatment Options

Of the 30 articles, patients also desired more information on treatment options in 6 (20%) articles [12,15,17,19,21,28]. Specifically, more information on the advantages and potential risks of various treatment options was needed [15,17,19]. The study conducted by Saroa and colleagues [17] found that 51.7% (n=106), 49.2% (n=101), and 46.8% (n=96) of the sample population (N=205) needed more information on how treatment works against the cancer, the evidence behind treatment recommendations, and how treatment is performed, respectively. During the COVID-19 pandemic, Yan and colleagues [37] found that patients wanted more information on HNC and COVID-19 and had concerns surrounding delays in cancer care due to pandemic precautions.

#### Nutrition

Of the 30 articles, 8 (27%) indicated that patients needed more information on nutrition [12,17,19,25,26,28,32,35]. In the study conducted by Saroa and colleagues [17], approximately half of the participants (n=205) wanted more information on managing diet to maintain nutritional intake. This finding was also stated by Manne and colleagues [26], with attention given to the difficulties associated with eating following treatment. Information was also needed on how to maintain weight during treatment and on feeding tubes [12]. Notably, a small study (n=11) found that participants were unsure of the necessity of referrals to nutrition services [35].

### Low Needs

#### Financial and Work Impact

Within the category of low-priority information needs, financial assistance and support was most frequently cited. Financial needs encompassed concerns about financial struggles and pressure [17,22,27], and obtaining information on sources of financial support [16,24,31,37]. Similarly, concerns surrounding the impact of HNC on patients’ “ability to work” were noted [12,17], as well as the need for resources regarding “support for returning to work” [31]. Although financial assistance was the most common low-priority need, 2 (6.7%) articles specifically listed obtaining insurance as a moderate-priority need [26,31]. Manne and colleagues [26] found that 33% (n=30) of participants (N=92) needed assistance obtaining health, life, or disability insurance following diagnosis.

#### Sexual and Oral Health

Sexual and oral health needs were also identified as low-priority needs. Of the 30 articles, 7 (23%) identified sexual intimacy as an information need among a limited subset of patients. Seven (23%) articles of varying sample sizes consistently found needs in this domain relating to psychosexual health after diagnosis and the impact of cancer on intimate relationships [12,23,25,26,28,29,31]. Similarly, information on fertility after treatment was noted in 2 (7%) articles. Fang and colleagues [28] reported that 1 patient, in their cohort of 65 patients, wanted more information on pregnancy following cancer treatment. The other, larger study found that 5.2% (8/154) of participants needed assistance with starting a family due to treatment or disease-related fertility struggles [31]. Regarding oral health, 3 (10%) articles found that patients desired more information on dental care, specifically on maintaining oral health [25,26,29].

### Needs Change Across Treatment Phases

Information needs were noted to be temporal, changing throughout the cancer trajectory. During the pretreatment phase, the education focus was on medical information, such as disease explanations, medical tests, and the basics of treatment modalities. Prognostic and disease information and explanation of medical tests were cited as high needs, with being informed on various treatment options as a moderate need [12,16,17,19-22,25,26,28]. Information on benefits and side effects of treatment was also needed in this phase [12,16,17,19,20,23-27]. Other information in this phase pertained to communication with health care professionals. Articles noted that patients and caregivers had moderate needs in relation to improving communication and care coordinating discussions [16,22,24,27,31,32,36,38]. Before and during treatment, caregivers needed information on how to support stress reduction for the patient [38]. Playing a role in treatment decisions and accessing appropriate medical care for the patient were also caregiver needs in this phase [24,38]. Needs in the financial domain were also noted in the pretreatment phase, with concerns relating to the impact of treatment on the ability to work and financial status [12,16,17,22,24,27,31,37].

During treatment, nutritional needs were prevalent as nutritional support and intervention is an integral part of the management of HNC [41]. Moderate concerns were noted relating to managing diet and weight. Study participants sought information on how to maintain their nutrition, as well as strategies to improve eating and drinking [12,17,19,25,28,32,35].

Needs shifted away from the medical domain in the posttreatment, survivorship phase and focused mainly on physical and psychological well-being. Studies consistently indicated that patients and caregivers desired more information on long-term care, such as physical changes, long-term side effects, and impact on QOL [17,18,22,25-27,31,33,34].

Of the 30 articles, nearly half (n=13, 43%) of the articles reviewed captured pretreatment needs during the posttreatment phase due to cross-sectional study designs. Retroactively, patients indicated that having more comprehensive information pretreatment could have supported treatment decisions [15,18]. Additionally, across 14 (47%) articles, information was needed on accessing supportive care services, strategies to manage stress and anxiety, body image concerns, and fears of recurrence [12,16,17,21,23,25-28,31,33,36,37]. Moreover, patients desired strategies on dealing with social situations following treatment [17,23,25,26,28,31], such as speaking and eating in public [12,17,19,23,26,28]. Information on lifestyle changes, like diet and exercise, to prevent recurrence was noted as a need [12,17,20,21,23,25,26,28]. Patients also wanted to learn about symptoms of recurrence, indicating that information on disease presentation is still needed after treatment [17,19,26]. Other needs in the survivorship phase were relevant to the impact of treatment on sexual intimacy [12,23,25,26,28,29,31,36] and fertility [28,31]. The ability to obtain insurance was also a need following diagnosis and treatment [26,31].

### Needs by Role

Three studies solely assessed the information needs of caregivers [16,24,38], while 2 studies investigated the needs of caregivers in conjunction with patients [37,40]; the remainder only considered the needs of the patient. Caregivers were conceptualized in these articles as family members.

Caregivers were also determined to have moderate and high-level needs that are unique from the patient. Needs were high for information on how to reduce stress for the patient [16,24,38], to play a role in treatment decisions, and to obtain the best medical care for the patient [24,38]. Moreover, caregivers also needed adequate information on the benefits, risks, and psychological effects of treatment to participate in collaborative decision-making regarding treatment options [16]. Among 102 caregivers, 90.2% (n=92) indicated they needed the opportunity to discuss concerns with the physician during pretreatment consultations [16,24].

Caregivers needed more information specific to their role [16,22,31]. One larger study (n=197) found that information on looking after the caregiver’s health, coping with fears about the patient’s deterioration, and how to balance care was a moderate need [24]. Additionally, caregivers wanted assistance in setting up family meetings with the patient’s care team, with this need being most prevalent at the time of diagnosis and treatment start [38].

### Design of Informational Tools

Only one study looked at the design of informational tools or specific formats in which the patient preferred to receive information [18]. Hoesseini et al [18] examined patient preferences and commented on the acceptability of visual schematics for prognostic information, comparing pie charts, bar charts, 100-person diagrams, survival graphs, and 100-square charts. The focus group (n=21) revealed that patients preferred to receive prognostic information in a pie chart as it was perceived as less confronting and clearer than the other formats. Patients expressed negative views of the 100-person diagram and the survival graph. Information presented in these formats was perceived as “too mathematical.” The study concluded that patients preferred to receive visual prognostic information in conjunction with a verbal explanation instead of a verbal explanation alone.

In our results, cancer information was provided through various modes (Table 3), with the most frequently cited source being verbal information from health care professionals, followed by written information from leaflets, pamphlets, and booklets, and information from the internet. Other sources of information included various peer groups, digital sources, books and magazines, and traditional media sources. Two articles indicated that patients received information from “other” sources [30,37].

**Table 3.** Used information sources.

Information source and article citation	Participants, n	Articles, n	Patients, n	Total participants, n
Internet		9	411	1363
Badr et al [23]	93			
Bisschop et al [42]	19			
Gibson et al [13]	21			
Inglehart et al [30]	235^a^			
Jabbour et al [12]	597			
Parker et al [27]	12			
Saroa et al [17]	205			
Watson et al [34]	10			
Yan et al [37]	171			
Peers				
Family		3	220	533
Badr et al [23]	93			
Inglehart et al [30]	235^a^			
Saroa et al [17]	205			
Friends or coworkers		2	148	328
Badr et al [23]	93			
Inglehart et al [30]	235^a^			
Other patients		2	26	205
Saroa et al [17]	205			
Taylor et al [40]	37^b^			
Support groups		1	18	205
Saroa et al [17]	205			
Verbal sources (from medical professionals)		15	1012	1115
Badr et al [23]	93			
Inglehart et al [30]	235^a^			
Jabbour et al [12]	597			
Saroa et al [17]	205			
Yan et al [37]	171			
Bisschop et al [42]	19^b^			
Brockbank et al [39]	24^b^			
Findlay et al [35]	11^b^			
Gibson et al [13]	21^b^			
Manne et al [26]	92^b^			
McQuestion and Fitch [33]	17^b^			
Parker et al [27]	12^b^			
Rhoten et al [14]	38^b^			
Taylor et al [40]	37^b^			
Watson et al [34]	10^b^			
Written sources				
Leaflets, pamphlets, or booklets		9	465	1056
Bisschop et al [42]	19			
Inglehart et al [30]	235^a^			
Jabbour et al [12]	597			
Saroa et al [17]	205			
Bozec et al [21]	46^b^			
Brockbank et al [39]	24^b^			
McQuestion and Fitch [33]	17^b^			
Taylor et al [40]	37^b^			
Watson et al [34]	10^b^			
Books		2	55	440
Inglehart et al [30]	235^a^			
Saroa et al [17]	205			
Magazines		1	19	235
Inglehart et al [30]	235^a^			
Medical journals		1	6	205
Saroa et al [17]	205			
Digital sources (CD or DVD)		3	46	597
Jabbour et al [12]	597			
Bozec et al [21]	46^b^			
Watson et al [34]	10^b^			
Media sources				
Television		2	19	440
Inglehart et al [30]	235^a^			
Saroa et al [17]	205			
Radio		1	9	235
Inglehart et al [30]	235^a^			
Newspaper		1	3	235
Inglehart et al [30]	235^a^			
Other (not specified)		2	31	406
Inglehart et al [30]	235^a^			
Yan et al [37]	171			

a Information seeking participants.

b Article did not provide exact number of participants that used the information source, so the number of participants is not accounted for in the total.

The preferred mode of delivery for information varied and reflected the age, sex or gender, and country of the sample populations. In the 12 (40%) articles where this question was investigated, three forms emerged as the preferred mode of delivery: (1) one-on-one meetings with health care professionals, as they allowed the opportunity to ask questions and gain immediate clarification [39]; (2) written and video materials, as patients could refer back to the materials when needed [28,39]; and (3) the internet, as it could provide specific information that may not be covered in other forms of delivery [42]. Having access to and using multiple modalities of information was preferred by patients in some studies [12,18].

## Discussion

### Principal Results

Through our scoping review, it is evident that the information needs of patients with HNC and their caregivers are not always met to a satisfactory level in our current model of information delivery. Additionally, patients and caregivers require information specific to the phase of treatment they are in. Without this, patients have described feeling “left in the dark,” resulting in decisional regret [43]. There is a need to provide patients and their caregivers with the latest information surrounding the impacts of surgery and radiotherapy so patients can make a confident decision, reducing decisional regret. Consequently, the design of new decision support tools should be considered to provide context-sensitive information relevant to patients’ specific information needs along the trajectory of the patient journey.

### Comparison With Prior Work

The literature has also revealed that there is a need for information that can support joint decision-making between the patient and the provider to improve patient satisfaction and outcomes. It has been shown that patient satisfaction is better when the patient is an equal member in their health care decisions [44]. The challenge for health care professionals is that QOL and health-related outcomes are not always easy to predict, so thorough communication of the implications of differing treatment approaches, risks, and benefits associated with treatments is crucial to reduce or prevent decision regret. Researchers have attempted to define characteristics of patients associated with poorer QOL or health-related outcomes. Linear mixed models were applied to examine changes over time and associated characteristics of 156 patients with HNC who completed repeat measures of physical and mental health over 5 time points from initiating radiation therapy to 6 months posttreatment [45]. The QOL deteriorated during radiation therapy and gradually improved after completion. Less social support was negatively associated with both physical and mental QOL. Older age, more comorbidities, more psychological symptoms, and concomitant chemotherapy were negatively associated with physical QOL. Male sex, fewer physical symptoms, surgery before radiation therapy, and concomitant chemotherapy were positively associated with mental QOL [45]. Future research should examine the relationship between treatments, patient characteristics, caregiver characteristics, and the role and nature of information needs in the development of information interventions to support patient decision-making, improve QOL, and reduce decisional regret.

There is a need to develop strategies to enhance shared decision-making to best facilitate patient-centered care. Barriers to shared decision-making have been identified as “uncertainty in the treatment decision, concern regarding adverse effects, and poor physician communication” [46]. Our ongoing work builds on the results of the current scoping review. Based on information needs reported in the literature, we are working on developing an electronic decision aid that will allow patients and caregivers to jointly explore decision options and their impact in collaboration with their health care providers. To date, few studies have examined the use of electronic decision aids and mobile apps in delivering information for supporting patient decision-making for HNC. In addition, there is very limited research discussing preferences for the mode of information delivery (eg, video, paper, or electronic tool). The aim is to support joint decision-making around HNC with the design of the Head and Neck Application for Patients and Their Partners (HANC APP) decision support tool. It is hoped that such a tool will help address a range of concerns to improve patient understanding of treatment options and their outcomes.

Lastly, managing side effects of treatments can lead to significant caregiver burden, yet caregiver information needs are underrepresented in the literature. “Ongoing assessment of caregivers’ support needs may contribute to enhancing the care and management of patients with HNC as well as helping patients and caregivers to develop effective coping mechanisms over time. Clinicians should view the HNC management and offer adequate support and education programs relevant to their needs” [47]. Collaborative conversation and planning may help reduce the dissonance in knowledge and what is considered “important” between oncologists, patients, and caregivers to ensure all information has been sufficiently covered, including topics that may not always be front of mind, such as toxicity management, transportation, and the facilitation of hydration to ensure proper care is provided for patients with HNC.

### Limitations

In considering the literature that was identified in our scoping review, a number of limitations can be identified that warrant further research. This included the finding of a few studies that have examined how to optimally design information for patients, with only one study commenting on the acceptability of visual schematics (eg, pie charts, person diagrams, and survival curves). In addition, a large proportion of the studies were cross-sectional, with pretreatment needs being measured in the posttreatment phase (as opposed to longitudinal studies). As such, the results of these studies relied heavily on patient recall and could have been influenced by survivor bias. Furthermore, since the publication of the studies that were identified, new surgical and radiation therapy techniques have appeared and continue to develop [48,49]. Finally, survival rates can be similar between different treatment modalities (eg, surgery and definitive chemoradiation therapy), but long-term toxicities vary [50-52]. As a result, decision regret is an emerging field of study. Thorough counseling in treatment choice is crucial and warrants future research in the area of HNC.

### Conclusions

Patients with HNC are demonstrated to have high information needs, which serve as prerequisites for making a fully informed decision on treatment options. Domains of high interest that are not adequately being met through standard of care are details on their specific type of cancer, medical tests and procedures, advantages and risks of available treatments, strategies to maintain eating and speaking, lifestyle guidelines for survivorship, and HPV transmission. Additionally, health care professionals must consider the evolving needs of the patient and address patient concerns on an individual basis to support patient autonomy and shared decision-making to the degree to which they are comfortable. The caregiver cannot be forgotten as they are demonstrated to also have unmet information needs. Tools are needed to support information delivery in a format that is resource-saving yet acceptable to patients. Implications of the review for the design of decision aids include the need for developing information tools that can provide context-sensitive information to better support patient understanding of risks, treatment options, and outcomes of choices. Such tools should support joint decision-making between the patient and the provider at different phases along the patient journey.

## Supplementary material

10.2196/64108 Multimedia Appendix 1 Full search strategy.

10.2196/64108 Checklist 1 PRISMA-ScR (Preferred Reporting Items for Systematic Reviews and Meta-Analyses Extension for Scoping Reviews) checklist.

## Abbreviations

HANC APP: Head and Neck Application for Patients and Their Partners
HNC: head and neck cancer
HPV: human papillomavirus
PRISMA: Preferred Reporting Items for Systematic Reviews and Meta-Analyses
PRISMA-ScR: Preferred Reporting Items for Systematic Reviews and Meta-Analyses Extension for Scoping Reviews
QOL: quality of life
