# Supplementary material for: Information Needs of Patients With Head and Neck Cancer and Their Supports in Relation to Treatment Management Choices: Scoping Review
Source: JMIR Cancer. 2025 Aug 21;11:e64108. doi: 10.2196/64108 (PMC12370262; doi:10.2196/64108)
Supplement: Multimedia Appendix 1 [file cancer-v11-e64108-s001.docx]

# Full Search Strategy

Embase <1974 to 2022 September 20>

1       *Patient Education/     30933
2       *Consumer health information/   1801
3       1 or 2  32636
4       *Information Dissemination/     5427
5       *Medical Information/   13238
6       *Information Service/   7215
7       exp *Teaching/  39466
8       exp *Access to Information/     3574
9       *Information Seeking/   1916
10      *Web-based Intervention/        1190
11      *Visual Information/    1699
12      *Publication/   44854
13      ((information* or knowledge or education*) adj3 (use* or using or utili* or seek* or search* or retriev* or transfer* or need* or want* or desire* or interest* or require* or prefer* or choice* or intervent* or service* or disseminat* or provide or provision or access* or search* or find* or understand* or insufficient* or gap or gaps or demand* or barrier* or deliver* or "look for" or gather* or collect* or acquir* or support* or share or sharing)).ti,ab.    827827
14      ((patient* or inpatient* or outpatient* or survivor* or caregiver* or famil* or spouse*) adj4 (instruct* or teach* or educat* or learn* or inform* or knowledge or communicat* or resource* or decision)).ti,ab.        471935
15      ((education* or information* or instruct* or intervention) adj4 (online or "on line" or virtual or "computer assisted" or "computer-assisted" or internet or website* or "web based" or "web-based" or pamphlet* or handout* or leaflet* or brochure* or publication* or print or printed or booklet* or video* or audio* or multimedia or cd or dvd or program* or session* or communicat* or source* or resource* or curricul* or "in person" or "in-person" or oral)).ti,ab. 317348
16      or/4-15 1474156
17      exp *Cancer Patient/    150212
18      *Patient Satisfaction/  27761
19      *Patient Preference/    6020
20      *Patient Participation/ 10871
21      *Caregiver/     27519
22      exp *Family/    160389
23      *Friend/        2740
24      *Cancer Survivors/      12729
25      (patient* or inpatient* or outpatient* or caregiver* or family or families or relative* or parent* or sibling* or "adult child*" or son or sons or daughter* or mother* or father* or in?law* orspouse* or husband* or wife or wives or partner* or "significant other*" or couple* or friend* or survivor* or "support person*" or "support people").ti,ab.    14641334
26      or/17-25        14679611
27      16 and 26       930688
28      3 or 27 945908
29      "head and neck cancer"/ or exp "head and neck carcinoma"/ or exp head cancer/ or exp jaw cancer/ or exp lip cancer/ or exp mouth cancer/ or exp neck cancer/ or exp nose cancer/ or exp orbit cancer/ or exp paranasal sinus cancer/ or exp pharynx cancer/ or exp salivary gland cancer/ or exp tongue cancer/ or exp tonsil cancer/   178192
30      (("head and neck" or head or neck or pharynx or pharyngeal or mouth or lip or oral or oropharyngeal or oropharynx or tonsil* or hypopharyngeal or hypopharynx or nasopharyngeal or nasopharynx or laryngeal or larynx or sinus* or paranasal or tongue or "salivary gland$" or "parotid gland*" or submandibular gland* or palate or palatal or palatine or gingiva* or trachea or tacheal) adj5 (neoplas* or cancer* or tumor* or tumour* or carcinoma* or adenocarcinoma* or sarcoma* or malignan* or oncolog*)).ti,ab,kf.    245154
31      29 or 30        293031
32      exp Drug Therapy/       3279958
33      exp Cancer Therapy/     995831
34      (th or su or rt or dt).fs.      6922827
35      ("clinical intervention$" or treatment* or therapy or management).ti,ab.        9503960
36      exp Chemotherapy/       778167
37      exp Antineoplastic Agent/       2595453
38      Antineoplastic Protocol/        714
39      (antineoplastic* or "anti-neoplastic*" or chemotherap* or polychemotherap* or chemoimmunoradiotherap* or chemoimmunotherap* or chemoradiation or chemoradiotherap* or cisplatin or platinol or platamin or neoplatin or cismaplat or cetuximab or erbitux or docetaxel or taxotere or docefrez).ti,ab.  846082
40      ((anticancer* or "anti-cancer*" or cancer or cytotoxic*) adj5 (drug* or agent*)).ti,ab. 200128
41      ((systemic or hormone or hormonal or endocrine or immune or targeted) adj (therapy or therapies)).ti,ab.        179671
42      exp Radiotherapy/       611321
43      (radiat* or radiother* or irradiat* or radiosurger* or radiochemotherap* or radioimmunotherap*).ti,ab.  980065
44      exp Immunotherapy/      279483
45      exp Biological Therapy/ 1937538
46      exp Molecular Targeted Therapy/ 52251
47      Monoclonal Antibody Therapy/    820
48      (immunotherap* or immunochemotherap* or immunochemoradiotherap*).ti,ab. 180232
49      exp Orolaryngology/     0
50      Preoperative Care/      44281
51      exp Surgery/    5471113
52      (otolaryngology or pre-operative or cryosurgery or dissection or excision or resection or laryngectomy or parotidectomy or pharyngectomy or cordectomy or tracheotomy or tracheostomy or glossectomy or hypopharyngectomy or maxillectomy or tonsillectomy or laryngopharyngectomy or surgery).ti,ab.   2392450
53      *Shared Decision Making/        3286
54      *Patient Decision Making/       2518
55      ("decision-making" or "decision making" or "decision support").ti,ab.   253686
56      (treatment adj (decision* or choice* or selection)).ti,ab.      60283
57      "informed choice*".ti,ab.       3744
58      or/32-57        17080135
59      28 and 31 and 58        7277
60      limit 59 to yr="2009 -Current"  6040
61      60 not ((exp animal/ or nonhuman/) not exp human/)      5979
62      61 not (exp juvenile/ not exp adult/)   5845
63      limit 62 to english language    5701

Ovid MEDLINE(R) and Epub Ahead of Print, In-Process, In-Data-Review & Other Non-Indexed Citations <1996 to September 16, 2022>

1              exp Patient Education as Topic/ 68404

2              Consumer health information/   4258

3              1 or 2     72094

4              Health Education/            35196

5              Information Dissemination/        18128

6              Health Communication/ 3084

7              Information Services/    9388

8              exp Teaching Materials/ 72808

9              Access to Information/  6981

10           Information Seeking Behavior/  3083

11           Computer Assisted Instruction/ 10210

12           Internet Based Intervention/      1008

13           Pamphlets/        2854

14           Health Knowledge, Attitudes, Practice/  117692

15           ((information* or knowledge or education*) adj3 (use* or using or utili* or seek* or search* or retriev* or transfer* or need* or want* or desire* or interest* or require* or prefer* or choice* or intervent* or service* or disseminat* or provide or provision or access* or search* or find* or understand* or insufficient* or gap or gaps or demand* or barrier* or deliver* or "look for" or gather* or collect* or acquir* or support* or share or sharing)).mp.                631125

16           ((patient* or inpatient* or outpatient* or survivor* or caregiver* or famil* or spouse*) adj4 (instruct* or teach* or educat* or learn* or inform* or knowledge or communicat* or resource* or decision)).mp. [mp=title, book title, abstract, original title, name of substance word, subject heading word, floating sub-heading word, keyword heading word, organism supplementary concept word, protocol supplementary concept word, rare disease supplementary concept word, unique identifier, synonyms]         319029

17           ((education* or information* or instruct* or intervention) adj4 (online or "on line" or virtual or "computer assisted" or "computer-assisted" or internet or website* or "web based" or "web-based" or pamphlet* or handout* or leaflet* or brochure* or publication* or print or printed or booklet* or video* or audio* or multimedia or cd or dvd or program* or session* or communicat* or source* or resource* or curricul* or "in person" or "in-person" or oral)).mp. [mp=title, book title, abstract, original title, name of substance word, subject heading word, floating sub-heading word, keyword heading word, organism supplementary concept word, protocol supplementary concept word, rare disease supplementary concept word, unique identifier, synonyms]         234354

18           or/4-17 1163298

19           exp Patients/     57639

20           Patient Satisfaction/       83506

21           Patient Preference/        10404

22           Patient Participation/     24647

23           exp Caregivers/ 45142

24           exp Family/         255343

25           Siblings/               11733

26           Parents/              61587

27           Spouses/             10382

28           Sexual Partners/               18284

29           Adult Children/ 1658

30           Friends/               6176

31           Cancer Survivors/            7709

32           (patient* or inpatient* or outpatient* or caregiver* or family or families or relative* or parent* or sibling* or "adult child*" or son or sons or daughter* or mother* or father* or in?law* orspouse* or husband* or wife or wives or partner* or "significant other*" or couple* or friend* or survivor* or "support person*" or "support people").mp. [mp=title, book title, abstract, original title, name of substance word, subject heading word, floating sub-heading word, keyword heading word, organism supplementary concept word, protocol supplementary concept word, rare disease supplementary concept word, unique identifier, synonyms]         9166390

33           or/19-32               9179016

34           18 and 33             694660

35           3 or 34  696846

36           exp Head/ and Neck Neoplasms/              2464

37           (("head and neck" or head or neck or pharynx or pharyngeal or mouth or lip or oral or oropharyngeal or oropharynx or tonsil* or hypopharyngeal or hypopharynx or nasopharyngeal or nasopharynx or laryngeal or larynx or sinus* or paranasal or tongue or "salivary gland$" or "parotid gland*" or submandibular gland* or palate or palatal or palatine or gingiva* or trachea or tacheal) adj5 (neoplas* or cancer* or tumor* or tumour* or carcinoma* or adenocarcinoma* or sarcoma* or malignan* or oncolog*)).mp.  184149

38           36 or 37                184149

39           exp Drug Therapy/          1073165

40           Treatment outcome/     1090105

41           exp Therapeutics/           3581038

42           (th or su or rt or dt).fs.   4623272

43           ("clinical intervention$" or treatment* or therapy or management).mp. 7387157

44           exp Combined Modality Therapy/             230946

45           exp Antineoplastic Agents/          875586

46           exp Antineoplastic Protocols/     128063

47           (antineoplastic* or "anti-neoplastic*" or chemotherap* or polychemotherap* or chemoimmunoradiotherap* or chemoimmunotherap* or chemoradiation or chemoradiotherap* or cisplatin or platinol or platamin or neoplatin or cismaplat or cetuximab or erbitux or docetaxel or taxotere or docefrez).mp.        754339

48           ((anticancer* or "anti-cancer*" or cancer or cytotoxic*) adj5 (drug* or agent*)).mp.         142776

49           ((systemic or hormone or hormonal or endocrine or immune or targeted) adj (therapy or therapies)).mp.                133544

50           exp Radiotherapy/          146360

51           exp Chemoradiotherapy/             19084

52           Radioimmunotherapy/  2893

53           Radiotherapy, Adjuvant/              23340

54           Heavy Ion Radiotherapy/              1228

55           Radiotherapy, High-Energy/        2679

56           Radiotherapy, Image-Guided/    3801

57           Whole-Body Irradiation/               5599

58           (radiat* or radiother* or irradiat* or radiosurger* or radiochemotherap* or radioimmunotherap*).mp.                722878

59           exp Immunotherapy/     211532

60           Oncolytic Virotherapy/  3650

61           Immunotherapy, Adoptive/         10604

62           exp Immune Checkpoint Inhibitors/         15073

63           exp Antibodies, Monoclonal/      192923

64           exp Molecular Targeted Therapy/             34635

65           (immunotherap* or immunochemotherap* or immunochemoradiotherap*).mp. 136463

66           Surgical oncology/           706

67           exp Orolaryngology/       0

68           Preoperative Care/         41429

69           exp Surgical Procedures, Operative/        2375078

70           (otolaryngology or pre-operative or cryosurgery or dissection or excision or resection or laryngectomy or parotidectomy or pharyngectomy or cordectomy or tracheotomy or tracheostomy or glossectomy or hypopharyngectomy or maxillectomy or tonsillectomy or laryngopharyngectomy or surgery).mp.               2282225

71           Decision Support Techniques/    20690

72           exp Decision Making/     187124

73           ("decision-making" or "decision making" or "decision support").mp. [mp=title, book title, abstract, original title, name of substance word, subject heading word, floating sub-heading word, keyword heading word, organism supplementary concept word, protocol supplementary concept word, rare disease supplementary concept word, unique identifier, synonyms]      262607

74           (treatment adj (decision* or choice* or selection)).mp.  35271

75           "informed choice*".tw. 2743

76           or/39-75               10464571

77           35 and 38 and 76              4307

78           limit 77 to yr="2009 -Current"    3224

79           78 not (Animals/ not (Animals/ and Humans/))   3213

80           79 not ((exp infant/ or exp child/ or adolescent/) not exp adult/) 3135

81           limit 80 to english language         3008
